# “It is great what we have learned from each other!” – Bedside teaching in interprofessional small groups using the example of Parkinson’s disease

**DOI:** 10.3205/zma001661

**Published:** 2024-02-15

**Authors:** Christine Schneider, Petra Anders, Thomas Rotthoff

**Affiliations:** 1 University Hospital Augsburg, Department of Neurology and Clinical Neurophysiology, Augsburg, Germany; 2 University Hospital Augsburg, Academy for Health Professions, Vocational School for Physiotherapy, Augsburg, Germany; 3 University of Augsburg, Faculty of Medicine, Medical Didactics and Educational Research (DEMEDA), Augsburg, Germany

**Keywords:** interprofessional, neurology, physiotherapy, vocational traning, medical students, bedside teaching, UWE-IP

## Abstract

**Background:**

While patient care often involves interprofessional collaboration, interprofessional teaching formats with participants from medical and physiotherapy fields are still rare. Furthermore, interprofessional education often takes place as separate courses and is not integrated into the clinical curriculum. Therefore, the goal of this project was to develop and implement interprofessional content into bedside teaching.

**Course development:**

The clinical subject of the course was “Parkinson’s disease”, as this condition allowed for the exemplary demonstration of interprofessional teamwork and different competencies. Through interprofessional bedside teaching and a specific clinical context, interprofessionalism was intended to be integrated and experienced as natural part of clinical practice. The bedside teaching was complemented with work in break-out groups and a lecture.

**Evaluation:**

The course was first conducted in the winter semester 2021/22. Participants were medical and physiotherapy students. Teaching teams were also interprofessional. A concurrent evaluation was carried out using the University of the West of England Interprofessional Questionnaire (UWE-IP) before and after course participation. UWE-IP scores in all sub-scales indicated a positive attitude, except for the “Interprofessional Learning” scale among physiotherapy students, which reflected a neutral attitude. Significant group differences were observed in the same scale at the pre-course time point between medical and physiotherapy students (p<0.01) and among medical students before and after course participation (p=0.02).

**Conclusion:**

The course proved to be well-suited for integrating interprofessional content into clinical education and can serve as a model for future teaching units. The evaluation reflected a positive attitude toward interprofessional learning.

## Zusammenfassung

**Hintergrund:**

Während Patientenversorgung häufig interprofessionell erfolgt, sind interprofessionelle Lehreinheiten mit teilnehmenden Personen aus Humanmedizin und Physiotherapie noch eine Seltenheit. Zudem findet interprofessionelle Lehre häufig immer noch in Form von gesonderten Veranstaltungen mit interprofessionellen Inhalten statt und sind nicht in das klinische Curriculum integriert. Ziel dieses Projekts war daher die Entwicklung und Implementierung eines interprofessionellen Unterrichts an Patient*innen (UaP).

**Kursentwicklung:**

Klinisches Thema des Kurses war „Morbus Parkinson“, da sich anhand dieser Erkrankung interprofessionelle Teamarbeit und unterschiedliche Kompetenzen exemplarisch darstellen ließen. Anhand eines interprofessionellen UaP und konkretem klinischen Kontext sollte Interprofessionalität als selbstverständlicher Teil des klinischen Handelns integriert und erlebbar gemacht werden. Eine Ummantelung des UaP erfolgte mit einer Vorlesung und eigenverantwortlicher Kleingruppenarbeit.

**Evaluation:**

Der Kurs wurde erstmals im Wintersemester 2021/22 durchgeführt. Teilnehmende Personen waren Medizinstudierende und Physiotherapieschüler*innen, die Dozierendenteams waren ebenfalls interprofessionell. Eine begleitende Evaluation erfolgte mit Hilfe des University of the West of England Interprofessional Questionnaire (UWE-IP) vor und nach Kursteilnahme. Im UWE-IP ergaben sich in allen Subskalen Summenscores, die einer positiven Einstellung entsprachen. Einzige Ausnahme war die Skala „Interprofessionelles Lernen“ bei Physiotherapieschüler*innen, die einer neutralen Einstellung entsprachen. Signifikante Gruppenunterschiede ergaben sich in derselben Skala zum Zeitpunkt vor Kursteilnahme zwischen Medizinstudierenden und Physiotherapieschüler*innen (p<0,01) sowie bei Medizinstudierenden vor und nach Kursteilnahme (p=0,02).

**Schlussfolgerung:**

Der Kurs erwies sich als gut geeignet, um interprofessionelle Lehrinhalte in die klinische Lehre zu integrieren und kann als Modell für weitere Lehreinheiten dienen. Die Evaluation spiegelte eine positive Einstellung bezüglich interprofessionellem Lernen wieder.

## 1. Introduction

### 1.1. Background 

Interprofessional collaboration in medicine is considered a crucial factor for quality control in patient care and for addressing the increasing demands within the healthcare system. Additionally, patient safety and work satisfaction in medicine can be enhanced through effective interprofessional collaboration [1], [2], [3]. The latter is characterized by knowledge of each other's competencies and roles, and a shared understanding of work tasks [4]. A prerequisite for this is an interprofessional education where students from at least two professions study together [5], [6], [7]. Interprofessional competencies are thus embedded in the graduate profile of the German National Competency-Based Learning Objectives Catalog for Medicine (NKLM 2.0) [8].

Previous studies indicate that at the beginning of their training, there is a high level of acceptance among students regarding interprofessional learning [9], [10], which significantly decreases over the course of their studies, along with a reduction in the number of interactions between students of different professions [10].

According to these findings, interprofessional teaching should ideally commence early in the academic program to capitalize on students’ positive attitudes. While interprofessional instructional sessions are now offered in various university locations, they often consist of individual, standalone events, usually in the form of voluntary elective courses [11], [12], [13], rather than being a standardized part of the curriculum [14], [15]. Interprofessional training wards during the final year of medical studies (practical year) are positioned at the end of the medical program and are also only available to a subset of students. Due to the practical nature of their training, physiotherapy students often have more frequent interactions with other healthcare professions. However, structured interprofessional teaching units are still the exception for them. Joint events for medical students and physiotherapy students have primarily been limited to anatomy courses [16], [17].

### 1.2. Objective 

The aim of this project was to develop and implement an interprofessional teaching concept, where students from two health professions were taught together in a clinical context by a multiprofessional team. This initiative was in response to the requirements of patient care and the resulting graduate profile of NKLM 2.0. in Germany. The goal was to make interprofessionalism tangible, based on the recommendations for designing interprofessional education from the Institute for Medical and Pharmaceutical Proficiency Assessment [9] and the Framework for the Development of Interprofessional Education at the University of Toronto [18]. By embedding the content as closely as possible in the clinical context, the intention was to avoid the perception of interprofessional content as purely academic and separate from clinical relevance. An interesting clinical theme was chosen to garner high acceptance for the subject and to engage learners who had so far limited exposure to interprofessionalism. The teaching units were integrated into the core curriculum of study and training, hence emphasizing their importance, rather than being offered solely as an elective, separate event on interprofessionalism. Lastly, the integration into the respective core curricula underscored the significance of interprofessionalism.

Concurrently, participants were to be evaluated before and after completing the teaching units regarding their attitudes toward various aspects of interprofessionalism.

## 2. Project description

### 2.1. Background 

The curriculum of the Medical Faculty Augsburg is designed as a competency-oriented, spiral curriculum with vertical and horizontal integration of subject areas [19]. From the first semester onwards, clincial content is interwoven with basic. For the general curriculum, continuous longitudinal learning objectives related to interprofessionalism were developed, aligning with NKLM 2.0 [8] and the Framework for the Development of Interprofessional Education at the University of Toronto [18]. These objectives were categorized into “collaboration”, “communication”, and “values and ethics”, and temporally into the axes of “exposure/introduction”, “immersion/development”, and “competence/practice”.

### 2.2. Project development and course implementation 

The teaching units were attended by medical students and physiotherapy students. They were taught by interprofessional teaching teams from the departments of neurology and physiotherapy. “Parkinson’s disease” was selected as clinical subjet, as this condition allows for the exemplary demonstration of interprofessional teamwork and different competencies. For instance, the competence “clinical examination” is applied from the neurological perspective to establish a diagnosis, and from the physiotherapeutic perspective to determine the therapeutic regimen. Consequently, the process of clinical examination differs, despite fundamental similarities, depending on whether it is conducted by a neurologist or a physiotherapist. The exemplary selection of Parkinson disease was further emphasized by the well-established close multiprofessional collaboration in the clinical setting.

In alignment with the integration of the course into the respective core curricula of both groups, “subject-specific” neurological and physiotherapeutic learning objectives, as well as interprofessional learning objectives, were formulated. The interprofessional learning objectives were embedded in the longitudinal learning objectives described in 2.1 and were established as follows:

After completing the course, students should be able to...

- describe the professional competencies and responsibilities of the involved professions,
- acknowledge shared responsibility for patient care,
- apply their profession-specific knowledge and collaborate to align priorities and requirements,
- justify the importance of interprofessional therapy using the example of Parkinson’s disease,
- present patients in a multiprofessional team.

**Figure 1:**
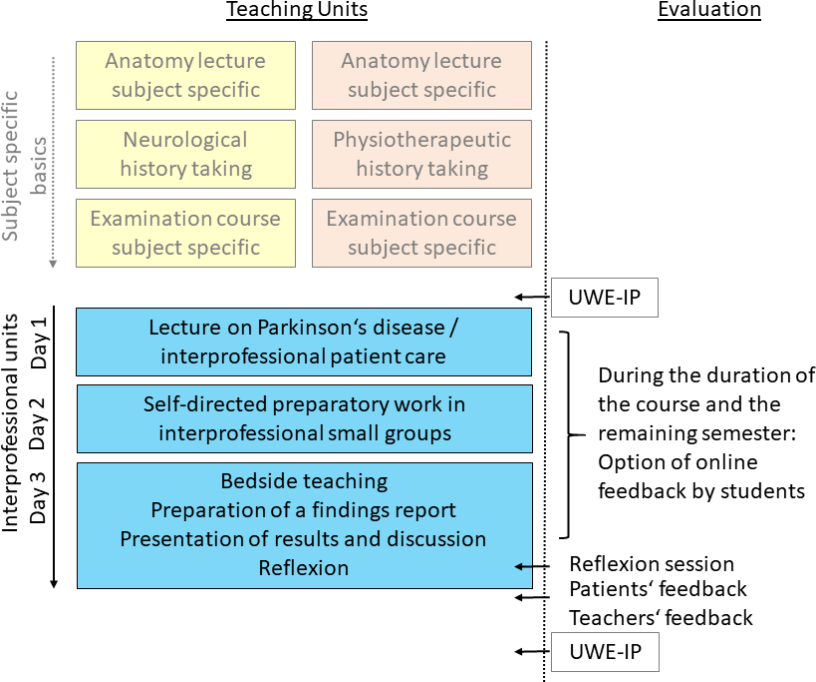
Schematic overview of teaching units and evaluation time points. Teaching units include an interprofessional lecture as the opening event on day 1 and break-out groups on day 2, followed by a reflective session in the small groups. In the right column, data collection using UWE-IP and open feedback are indicated. Additionally, the integration into the general curriculum in the third semester/second year is illustrated, when both professions are taught neuroanatomy, medical history, and neurological examination.

A schematic overview of the teaching units is found in figure 1 (Fig. 1). They took place in the third semester of medical studies and the second year of physiotherapy training, at a time when both groups focused on neuroanatomy. In the third semester, medical students also had a session on neurological history-taking and their first neurological examination course. For the physiotherapy students, sessions on gait and balance, including specific history-taking and examination, were scheduled, allowing both groups to draw on a relatively broad base of prior knowledge.

Bedside teaching itself was prepared with a joint lecture for all participants, providing knowledge on Parkinson’s disease and principles of disease-specific history-taking, physical examination, and interprofessional therapy. It was designed by an interprofessional team of lecturers from the fields of neurology, physiotherapy, and speech therapy.

As further preparation, students, in interprofessionally mixed break-out groups, researched common and clinically relevant symptoms of Parkinson’s disease and possible pharmacological and non-pharmacological forms of therapy. Additionally, they reflected on the prerequisites of meaningful, patient-centered interprofessional communication. Groups had the flexibility to meet for this asynchronous part either self-organized online or in person. They used guiding questions for their research task, which were made available through the online learning platform.

The central teaching unit, and simultaneously the conclusion of the course, were the bedside teaching lessons, when all the previously acquired knowledge could be applied. In interprofessionally mixed small groups, students took histories and conducted neurological or physiotherapeutic examinations on Parkinson's patients. Subsequently, each group summarized their history and examination findings in a shared document. They then identified the symptoms, which seemed relevant for treatment, and assigned them to profession(s) for possible treatment. The goal was to create awareness of the professions involved, without creating a detailed therapy plan, which would not have been feasible for students at this stage. The results were presented in a plenary session afterwards. Following feedback from teaching teams on communication with patients and team communication, on data collection, and on presentation, the competencies and limitations of the involved professions were discussed. At the end, there was time for self-reflection, reflection on teamwork, and on the interprofessional content taught. In the form of a “flashlight” feedback, all participants (students, trainees, patients, and instructors) where asked on open-ended questions: “What will you take away from this course? What can we do better?”, which was followed by further discussion based on these answers.

### 2.3. Participants 

As explained in 2.2, the courses were attended by medical students in the third semester (n=84) and physiotherapy students in the second year of training (n=20). The courses were integrated into the core curriculum for medical students (not an elective), although attendance was not mandatory. For the physiotherapy students, attendance was compulsory.

### 2.4. Evaluation 

Concurrently with the implementation, the course was evaluated. This occurred, on one hand, in an informal manner through a “flashlight” feedback session at the end of the course. On the other hand, a standardized evaluation was conducted using the University of the West of England Interprofessional Questionnaire (UWE-IP) [20], a self-assessment questionnaire with four subscales (communication and teamwork, interprofessional learning, interprofessional interaction, interprofessional relationships), comprising a total of 35 items. The items are rated on a Likert scale from 1 (“I fully agree”) to 5 (“I strongly disagree”), except for the “communication and teamwork” subscale, which is rated from 1 (“I fully agree”) to 4 (“I strongly disagree”). The UWE-IP is available in a validated German translation and is widely used in longitudinal and cross-sectional surveys on interprofessional education [14], [21], [22], [23]. Data collection using the UWE-IP was carried out anonymously online before and after participation in the course.

In addition, data on age, gender, group affiliation (medical/phyiotherapy), and additional training in another health profession were collected. For the analysis, negatively formulated questions of the UWE-IP were recoded according to the guidelines. One missing answer per subscale was accepted for analysis. The statistical analysis was performed using the Statistical Package for Social Sciences (SPSS). Due to the small group sizes, group comparisons were conducted using non-parametric tests.

In addition to the standardized evaluation and the reflection session following the bedside teaching, students and trainees had the opportunity to provide anonymized feedback through the online-based learning platform. After the completion of the teaching sessions, instructors and patients with Parkinson’s were individually asked for free verbal feedback, which was documented in bullet points in an anonymized manner.

## 3. Results

### 3.1. Demographics 

The course, as described here, was conducted and evaluated for the first time in the winter semester 2021/22. Nearly one-third (32%) of medical students and 100% of physiotherapy students participated in the first evaluation, taking place before course participation. The course was completed by 66 medical students (75% of the semester) and 18 physiotherapy students (90% of the cohort). Of these, 82% of medical students and 72% of physiotherapy students participated in the second evaluation. Demographic data is presented in table 1 (Tab. 1). 32% (survey 1) and 30% (survey 2) of participating medical students already had training in another health profession, while this did not apply to any physiotherapy student.

### 3.2. Feedback

Feedback from participants was almost exclusively positive (such as the quote in the title of this work). Common learning from the beginning, making new contacts, and gaining a perspective “beyond one’s own field” were repeatedly mentioned as enriching. Both medical and physiotherapy students stated, that they had learned from each other through the course. Examples included different or additional physical examination techniques, such as the diverse assessment of gait, the clinical examination of different forms of tremor (not taught in physiotherapy classes), or the examination of rigidity of the trunk, which was unfamiliar to medical students. Physiotherapy students also mentioned acquiring additional knowledge about Parkinson’s disease. Some participants described the organization of self-directed small-groups in preparation for the bedside teaching as challenging. Deliberately, no specific date or location was provided, allowing for meetings in informal settings. The desire for further joint teaching units was repeatedly voiced. According to instructors (n=5), participants interacted with each other openly and respectfully. The topic of Parkinson’s disease effectively demonstrated the competencies and responsibilities of the involved professions. The differences in the clinical examination of gait and balance highlighted the associated competencies: while the (medical) neurological examination sought clues for clinical cardinal symptoms like hypokinesia and postural instability to diagnose the condition, the physiotherapeutic gait examination identified functional limitations more extensively, guiding therapy needs. Feedback from patients (n=8), most of whom were recruited through the regional Parkinson’s disease support, was also positive. They emphasized the opportunity to report various aspects of Parkinson’s disease, including psychological symptoms and difficulties in coping with daily life. All participating patients viewed a joint neurology and physiotherapy teaching session as sensible. As most of them received physiotherapy regularly, sometimes multiple times a week, they could contribute their experiences regarding the interfaces between health professions, and vividly describe the added value of multiprofessional therapy in terms of mobility and independence in daily life. However, experiences of inadequate coordination in the outpatient sector were also reported, typically based on a medical prescription and a physiotherapeutic assessment report, providing learners insights into the healthcare system.

### 3.3. UWE-IP 

#### 3.3.1. Sum scores 

The means and standard deviations of the UWE-IP survey are presented in table 1 (Tab. 1). In both surveys before and after course participation, medical students and physiotherapy students both achieved sum scores in each subscale that corresponded with a positive attitude toward the queried topics. Only in the “interprofessional interaction” subscale did the sum scores of physiotherapy students, both before and after course participation, correspond with a neutral attitude (Mean±SD 23.7±3.1 and 24.8±3.5, respectively), closely bordering on a positive attitude (cut off 22/23 points).

#### 3.3.2. Group differences 

Significant cross-sectional group differences between medical and physiotherapy students at the same time point only occurred in the “interprofessional learning” subscale at the time before course participation, with both groups scoring values corresponding with a positive attitude. After course participation, the group difference was no longer significant, and there were no significant differences in the other subscales between the two professions. In the longitudinal survey, the mean sum score of the ’interprofessional learning” subscale for the group of medical students significantly decreased before and after course participation. No other significant longitudinal group differences were observed (see table 1 (Tab. 1)).

## 4. Discussion

The teaching concept proved to be well-suited for conducting bedside teaching in an interprofessional setting, thereby integrating interprofessional teaching into the clinical context. The combination of physiotherapy and neurology was particularly effective in highlighting differences in tasks and competencies of each profession through fundamentally similar approaches in clinical examination. The introduction with preceding lecture and small group work allowed for a solid knowledge base before the start of bedside teaching, not only regarding Parkinson’s disease itself, but also concerning interprofessional threrapy. Additionally, various perspectives on the topic “Parkinson’s disease” could be presented in advance, enabling students to commence bedside teaching with an expanded view. From the instructors’ perspective, the greatest value of these preceding units was that students got to know each other, breaking down any potential barriers. This allowed participants from both professions to confidently take part in the bedside teaching from the start and to make the most of their time together.

The evaluation using UWE-IP showed a positive attitude towards the queried interprofessional content in all subscales for both groups – with the exception of the “interprofessional interaction” subscale for physiotherapy students. Similar to previous surveys using UWE-IP, our study demonstrated a positive attitude toward interprofessional teamwork, teaching, and relationships [14], [21], [22]. However, in our evaluation, medical students also exhibited a positive attitude toward interprofessional interaction, while two other studies reported negative values in this subscale [14], [22]. These previous results were independent of an extended stay in an interprofessional training ward [22] and irrespective of the stage of education – students were surveyed in the first [14] and final [22] years of their studies.

Limitations of this study included different group sizes, with the size of the physiotherapy student group dictated by the size of their class. The low participation of medical students in the first part of the evaluation and significant increase in the second part could also potentially bias the results. The low participation in the initial evaluation may be attributed to online recruitment and the limited exposure to the topic of interprofessionalism, that medical students had at this point of their training. In contrast, physiotherapy teachers actively advertised participation in the survey during their classes. Also, physiotherapy students already had some interprofessional contacts during the practical part of their training, which likely contributed to their 100% participation rate in the initial survey. Despite these limitations, the study reproduced results from previous interprofessional studies involving medical students and nursing trainees. One factor contributing to the positive attitude of our medical students toward interprofessional interaction could be the relatively high percentage of students with previous experience in another health profession, a characteristic influenced by the allocation of places in study course. Given the small group size, a subgroup analysis was deliberately avoided.

## 5. Conclusion

In summary, interprofessional education was successfully integrated into the clinical training of medical students and physiotherapy students using bedside teaching. The course was conducted repeatedly in the described format and proved to be easily sustainable. It can serve as a model for other interprofessional bedside units. In our view, any clinical condition, which is managed interprofessionally in daily practice and which involves similar (diagnostic) techniques applied by various professions would be suitable as topic. Examples could include conditions from the fields of trauma surgery or rheumatology combined with physiotherapy, or otolaryngology combined with speech therapy. The preceding interprofessional teaching units, as detailed above, were crucial for the success of the bedside teaching, and we recommend incorporating them into future events despite the additional effort.

## Acknowledgements

The authors thank all patients who volunteered for the teaching units. Special thanks go to Mrs. Böck, the head of the Augsburg regional group of the German Parkinson Association, for her vigorous support. The authors also thank Mrs. Anna Schmidt (School of Physiotherapy, University Hospital Augsburg) and Dr. Hildegard Kroiss (Department of Neurology and Clinical Neurophysiology, University Hospital Augsburg) for their support in planning and conducting the course units. The German version of UWE-IP was provided by the Department of General Medicine and Health Services Research at Heidelberg University Hospital, Heidelberg, Germany.

## Author’s ORCID

Thomas Rotthoff: 0000-0002-5171-5941

## Competing interests

The authors declare that they have no competing interests. 

## Figures and Tables

**Table 1:**
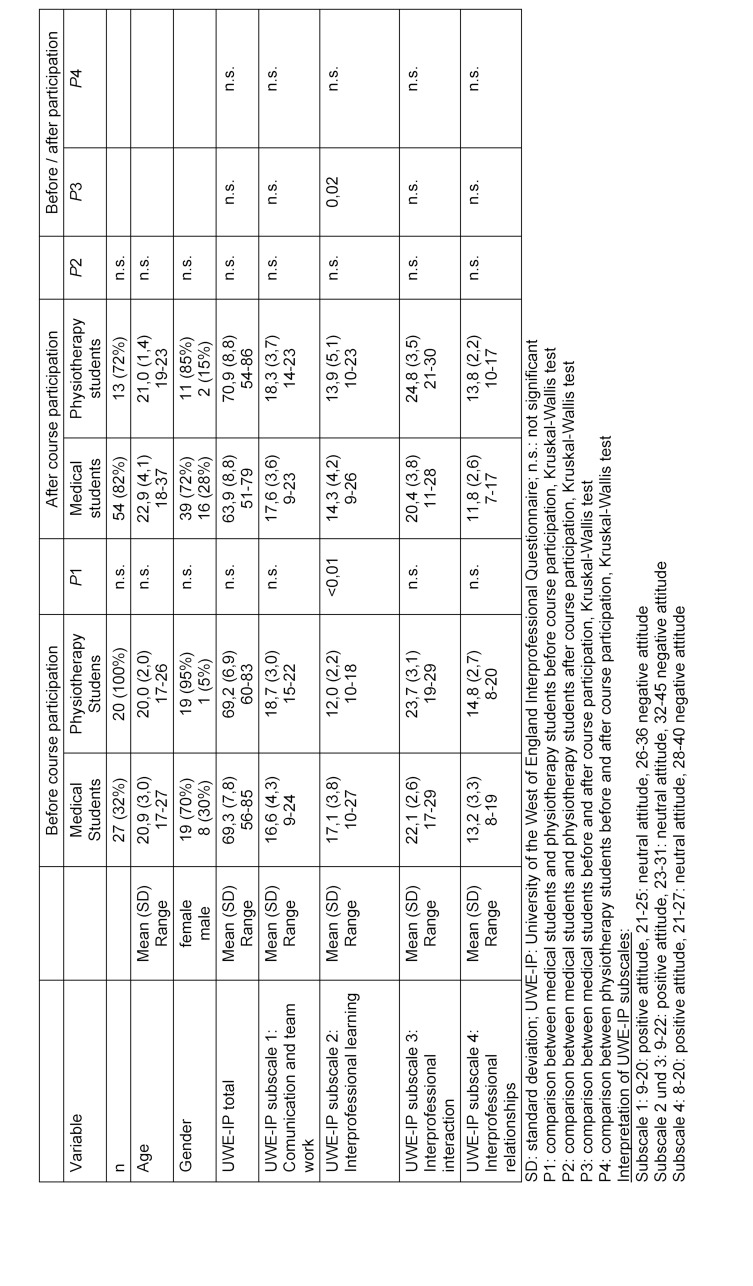
Demographic data and UWE-IP sum scores before and after interprofessional coure participation
